# Establishment of *Wolbachia* infection in *Aedes aegypti* from Pakistan via embryonic microinjection and semi-field evaluation of general fitness of resultant mosquito population

**DOI:** 10.1186/s13071-022-05317-4

**Published:** 2022-06-06

**Authors:** Muhammad Sajjad Sarwar, Nusrat Jahan, Azeem Ali, Hafiz Kamran Yousaf, Iqra Munzoor

**Affiliations:** 1 Department of Zoology, University of Okara, Okara, 56300 Pakistan; 2 Department of Zoology, Government College University, Katchery Road, Lahore, Pakistan; 3 Department of Statistics and Computer Science, University of Veterinary and Animal Sciences, Lahore, Pakistan

**Keywords:** *Wolbachia*, *Aedes aegypti*, *Aedes albopictus*, Embryonic microinjection, Cytoplasmic incompatibility

## Abstract

**Background:**

Dengue is a mosquito-borne viral disease that is mainly spread by *Aedes aegypti*. It is prevalent on five continents, predominantly in tropical and sub-tropical zones across the world. *Wolbachia* bacteria have been extensively used in vector control strategies worldwide. The focus of the current study was to obtain a natural population of *Ae.* *aegypti* harbouring *Wolbachia* and to determine the impact of this bacteria on the new host in a semi-field environment.

**Methods:**

*Wolbachia*-infected *Aedes* *albopictus* was collected from the city of Lahore, Punjab, Pakistan, and *Wolbachia* were successfully introduced into laboratory-reared *Ae. aegypti* via embryonic microinjection. The stable vertical transmission of *w*AlbB in the host population was observed for eight generations, and the impact of *Wolbachia* on the general fitness of the host was evaluated in semi-field conditions.

**Results:**

In the laboratory and semi-field experiments, *w*AlbB *Wolbachia* presented a strong cytoplasmic incompatibility (CI) effect, evidenced as zero egg hatching, in crosses between *Wolbachia*-infected males and wild (uninfected) females of *Ae. aegypti*. *Wolbachia* infection had no noticeable impact on the general fitness (*P* > 0.05), fecundity, body size (females and males) and mating competitiveness of the new host, *Ae. aegypti*. However, there was a significant decrease in female fertility (egg hatch) (*P* < 0.001). In addition, under starvation conditions, there was a remarkable decrease (*P* < 0.0001) in the life span of *Wolbachia*-infected females compared to uninfected females (4 vs. > 5 days, respectively).

**Conclusions:**

*Wolbachia* strain *w*AlbB has a great potential to control the dengue vector in *Ae. aegypti* populations by producing 100% CI with a limited burden on its host in natural field conditions. This strain can be used as a biological tool against vector-borne diseases.

**Graphical Abstract:**

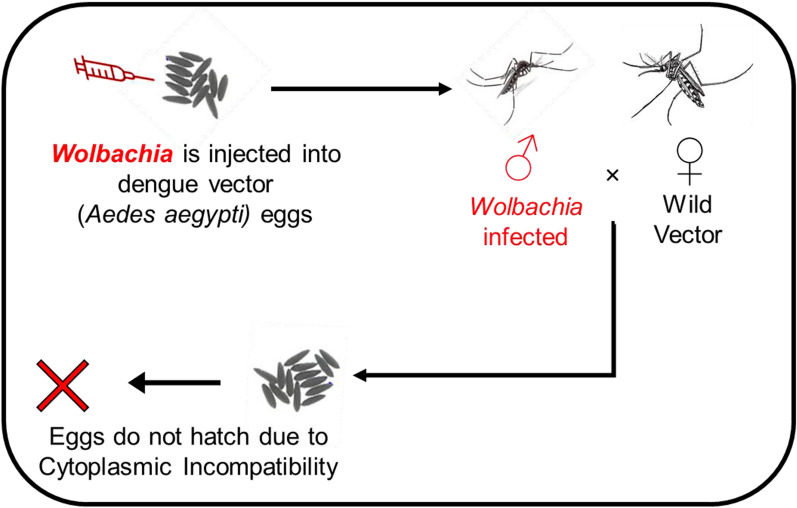

**Supplementary Information:**

The online version contains supplementary material available at 10.1186/s13071-022-05317-4.

## Background

*Aedes aegypti* is a mosquito of medical importance as it spreads dengue virus (4 serotypes) to millions of people worldwide annually. Pakistan has experienced the reoccurrence of dengue outbreaks during the last two decades. According to the WHO, 102,404 dengue cases and 278 deaths were reported in Pakistan within a 3-year period (2019–2021) [1]. To control dengue vectors, mainly* Aedes aegypti* mosquitoes but to a lesser extent* Aedes albopictus* mosquitoes, insecticides are imported into the country as a cost of billions of rupees annually. In addition to the high cost, the use of traditional insecticides is associated with many negative effects on the environment and communities. In the absence of a vaccine or antiviral drug against dengue virus, suppressing or replacing the vector population through novel methods are important approaches for disease control. One such approach is the use of the *Wolbachia*, a Gram-negative alpha-proteobacteria, which is now being used in various countries as a biological agent to control the progression of various vector-borne diseases, including dengue [2–6].

*Wolbachia* is a bacterium that is naturally present in many invertebrate species, particularly in nematodes and various arthropods, including insects, such as termites and springtails [7–11]. *Wolbachia* causes several reproductive modifications, such as cytoplasmic incompatibility (CI) [12], parthenogenesis, male-killing [13] and feminization, in their hosts [14]. Of these modifications, CI is the most common phenomenon, present in a wide range of insects that fail to complete karyogamy, conceivably by delaying nuclear envelope breakdown and mitosis. This mechanism may promote *Wolbachia* invasion of uninfected populations because infected females can mate and produce offspring successfully with both infected and uninfected males, whereas uninfected females are unable to produce offspring when they mate with *Wolbachia*-infected males [15].

*Aedes aegypti* lacks natural *Wolbachia* infection and, consequently, it may be artificially infected with *Wolbachia* naturally occurring in insects to utilize the unique features of this bacterium [16]. Different strains of *Wolbachia* can be used to control a particular disease through manipulation of the biology of the insect host in various ways, such as vector population suppression, direct interference with the transmission of pathogens to humans and negative effects on the fitness of different hosts (e.g. fecundity, fertility, larval development and longevity of mosquito vectors) [17–23]. In the last two decades, various laboratory and field experiments had been conducted with the aim to evaluate numerous strains of *Wolbachia* within mosquito vectors.

*Aedes* *albopictus* is naturally infected with two strains of *Wolbachia*: *w*AlbA and *w*AlbB [24]. The application of the proposed strategy requires transfection of a suitable *Wolbachia* strain through microinjection. The transfer can be carried out embryonically, by microinjection the cytoplasm of the *Wolbachia*-infected embryo of the donor insect (mosquitoes and fruit flies, etc.) to the recipient [25]. Artificial transinfection of *Wolbachia* strain from the native host (*Culex* or *Drosophila*) to another distantly related new host can be challenging [25–29]. Since the effect of different strains is highly variable on the hosts, however, the best strain for vector control has complete maternal transmission, maximum CI induction, low fitness cost, strong virus blocking ability and high occurrence under field conditions [30–33]. *w*AlbB was first introduced into *Ae. aegypti* in 2005, and was found to induce CI [29]. This strain inhibits dengue and other viruses from being transmitted by *Ae. aegypti* [34]. Moreover, *w*AlbB is more heat resistant than other *Wolbachia* strains [35], and *w*AlbB has been released in field trials, successfully reducing dengue transmission [36].

Given this background, we selected wild *Ae. albopictus* as a donor of *Wolbachia w*AlbA and *w*AlbB strains for transinfection to local *Ae. aegypti* mosquitoes collected from Lahore, Pakistan. It was expected that these *Wolbachia w*AlbA and *w*AlbB strains would be better adapted to the local warm environment and would have a better chance of inducing CI, spreading in the wild mosquito population and blocking the transmission of viruses. The fitness of the transinfected mosquito population was also evaluated in the semi-field conditions to obtain data allowing a better prediction in field conditions. This study provides baseline data for the experimental release of *Wolabchia*-infected dengue-resistant mosquitoes in the specific study area of population suppression and replacement.

The present study is designed to transfect the local strain of *Wolbachia* and investigate its effects on the local population of the host *Ae. Aegypti.* The objectives involve the transfection of *Wolbachia* from *Ae. albopictus* collected in Pakistan into the local *Ae. aegypti* population via embryonic microinjection and semi-field evaluation of the impact of *w*AlbB on the general fitness of the host population through the assessment of fecundity, fertility, larval to pupal development, CI induction potential, male competitiveness and life span.

## Methods

### Field collection and rearing of mosquito strains

A donor of *Wolbachia*, *Ae. albopictus*, and recipient, *Ae. aegypti* (hereafter referred to as “RAG”), adult mosquito populations were locally collected from Lawrence Garden, Lahore Pakistan (31°33′17.9″ N, 74°19′44.4″ E) in 2015 using a CDC backpack mosquito aspirator (model 1412; John W. Hock Co., Gainesville, FL, USA). Geographical coordinates were collected as DMS (degrees, minutes, seconds) using a GPS apparatus (model 76CSx; Garmin GPSMAP® USA, Olathe, KS, USA). Both populations were reared separately in an insectary at Govt. College University, Lahore, Pakistan, at 27 ± 0.5 °C ambient temperature and 80 ± 5% relative humidity, under a photoperiod of 12/12-h light/dark with 30 min of gradual transition of light as per standard rearing procedures [37]. Females aged about 5–6 days were blood-fed on defibrinated sheep blood through a membrane feeder for 20 min. Eggs were incubated for a minimum of 1 week.

### Detection of *Wolbachia* in *Ae. albopictus*

The presence of *Wolbachia* in *Ae. albopictus* was confirmed by PCR and then the transfection experiments were performed. Dissection of the reproductive organs of the field-collected *Ae. albopictus* and genomic DNA extraction and quantification were done as described by Sarwar et al. [38]. The extracted DNA (template) was exponentially amplified in a Techne Progene PCR thermal cycler (Marshall Scientific, Hampton, NH, USA) in a total reaction volume of 50 µl containing 1× Taq buffer, 1.5 mM MgCl_2_, 0.2 mM dNTPs, 0.4 µM each primer, 1 U *Taq* DNA polymerase and approximately 50 ng of DNA. Genomic DNA of RAG and *Culex quinquefasciatus* were used as the negative and positive control, respectively. Details on the general primer of *Wolbachia* (*Wolbachia* surface protein [*wsp*]) along with PCR conditions are given in Additional file 1: Figure S1a. The amplified products were then analysed by gel electrophoresis as reported by Sarwar et al. [39]. The presence of double infection of *Wolbachia* strains was also tested using *w*AlbA and *w*AlbB strain-specific *wsp* gene primers [40]. Details on these procedures are given in Additional file 1: Figure S1b, c.

### Embryonic microinjection for *Wolbachia* transfection

The microinjection protocol was adapted from Xi et al. [29]. Micropipettes (length: 10 cm) were prepared from Quartz tubing filaments (outside and inside diameters: 1.0 and 0.70 mm, respectively) using a laser-based micropipette puller (model PMP-102Q; MicroData Instrument, South Plainfield, NJ, USA). The sharp tip was then mechanically ground using MicroData Instrument’s Microelectrode Beveler (model MFG-5AP) to create the bevelled surface of the tip. For the microinjection, 10 blood-fed donor and recipient females were allowed to oviposit separately for about 60–90 min. The grey-coloured eggs were selected and aligned on a slide. The eggs of RAG mosquitoes were desiccated for a short period and then protected by a drop of halocarbon 700 oil (Sigma-Aldrich Co., St. Louis, MO, USA) to avoid further desiccation. Similarly, the *Ae. albopictus* eggs were aligned on the slide but without desiccation. In total, 376 RAG eggs were microinjected in four experimental groups. The injected embryos were considered to be filial generation zero (F_0_) and incubated in the insectary for 1 week, ultimately developing into adults.

The establishment of and screening for *Wolbachia* infection were carried out as described by Xi et al. [29]. Briefly, the first filial generation (F_1_) eggs of *Wolbachia*-positive F_0_ females of *Ae. aegypti* were reared (the transfected mosquito line is hereafter referred to as “WAG”), and all remaining (*Wolbachia*-uninfected) F_1_ eggs were discarded. The F_1_ females were separated at the pupal stage to keep them virgin and allowed to mate with RAG (uninfected) males in a 1:1 ratio. After mating, 5- to 6-day-old F_1_ females were blood fed, and F_1_ individual females were isolated and allowed to oviposit. Following oviposition, F_1_ females were also tested for *Wolbachia* infection using the PCR assay. Those F_1_ females of WAG that tested negative for the presence of *Wolbachia* were discarded along with their progeny. The F_1_ females carrying a double infection of *Wolbachia* strains (*w*AlbA + *w*AlbB) were selected to establish a *Wolbachia*-infected *Ae. aegypti* line. A maximum of 30 virgin WAG females were outcrossed with 30 RAG (uninfected) males (at a 1:1 ratio) for up to four generations to decrease genetic bottleneck effects in the WAG line [41]. The egg hatching rate of WAG was compared with that of RAG and the graph was plotted.

### Confirmation of *Wolbachia* infection in WAG at F_5_

A total of 15 virgin females and males were randomly selected from the WAG F_5_ stock line. Whole genomic DNA was extracted from the dissected ovaries of WAG. Double infection of *Wolbachia* strains was screened for by PCR, using the same procedure as mentioned above (for details, see Additional file 1: Figure S1b, c).

### Generation of aposymbiotic line

The aposymbiotic line was generated by removing *Wolbachia* infection from about 50 WAG mosquitoes at the F_5_ generation. The adults were fed a 10% sugar solution containing tetracycline solution at 1 mg/ml, pH 7 (Sigma-Aldrich; catalogue #T7660-5G) for 5 days per week for two consecutive generations to observed the impact of *Wolbachia* on the *Ae. aegypti* host. The mosquitoes were transferred from the stock cage to new cages using a handheld mechanical aspirator (model 2809 A; BioQuip Products Inc. Compton, CA, USA). The removal of *Wolbachia* was confirmed by the PCR, as mentioned above, in subsequent generations. This aposymbiotic line is referred to hereafter as “TWAG”.

### General fitness of WAG in the semi-field conditions

Semi-field evaluation was carried out from August to October 2016 in the GCUL Botanic Garden, Lahore (31°33′24.9″ N, 74°19′38.4″ E) to determine the effect of *w*AlbB *Wolbachia* at the F_8_-F_9_ generations on reproductive fitness (female fecundity and fertility), the time required for larval development, mosquito body size, mating competitiveness of WAG males to wild males, life span and degree of CI induction. All the semi-field experiments were done in triplicate independently.

The field cage was made up of a rounded rectangular shaped mosquito net (2.25 × 1.25 × 1.00 m). A two-cage design was employed to reduce the potential for accidental escape of laboratory-reared mosquitoes or the accidental introduction of wild mosquitoes (Additional file 1: Figure S2). Thus, the field cage itself was covered with a larger mosquito bed net on all sides and over the top. The field cage unit was kept on a wooden platform with the legs of the platform in water-filled bowls, and placed under a tree canopy *Alstonia scholaris* with climbing shrub *Vallaris solanacea*. However, as an additional protection from extensive sunlight and rainfall, a canvas tarpaulin (3 × 5 m) was suspended over each cage at a height of 2 m. The cage was provided with a flowerpot as a resting area and containers of a 10% sugar solution. Four semi-field cages were installed in the same environment. Environmental parameters, including temperature, relative humidity, light intensity and rainfall were recorded using a data logger at set intervals of 1 h, and mean values of each day were plotted.

#### Female fecundity and fertility

The average number of eggs laid per female (fecundity) was estimated in the RAG, WAG (at F_8_) and TWAG groups of mosquitoes. In the semi-field cages, one hundred 5- to 6-day-old gravid females of each group were transferred to twenty 50-μl Falcon tubes, five females per tube, and allowed to lay eggs. The egg hatching rate (fertility) of the three groups was also evaluated. After day 7 of incubation, the egg strips of each group were immersed in deoxygenated water, and the hatching rate was scored at 48-h post immersion. To see the effect of *Wolbachia* on oviposition, we used analysis of variance (ANOVA) to compare the difference in the means of all pairs. The proportion of egg hatching was tested using a Chi-square test of association. We proposed three hypotheses regarding the equality of: (1) RAG and WAG; (ii) WAG and TWAG; and (iii) TWAG and RAG; these were tested against the alternative hypotheses of no equality.

#### Larval development and wing length measurement

Post egg hatching, 100 larvae of each group were transferred to rearing pans. An equal amount of larval food (6% liver powder) was given to all groups daily. Pupae formation was recorded at 12-h intervals. A test of association was applied to days and pupae formation. The test hypothesis states that the number of days required for pupae formation and the number of pupae are independent. The alternative hypothesis states that these are not independent and that the number of days required for pupae formation and the number of pupae are associated. Wing length/area was considered to be an estimate of body size [42]; the latter has a strong impact on the fecundity of female and male mosquitoes. Wing length was measured as previously described by Joshi et al. [41]. The results of wing length measurement were analysed using the Wilcoxon–Mann–Whitney test.

#### Cytoplasmic incompatibility

To determine the ability of WAG to induce CI, four types of crosses in three biological replicates were designed between RAG and WAG F_9_ mosquito strains (RAG♀ × RAG♂, RAG♀ × WAG♂, WAG♀ × RAG♂ and WAG♀ × WAG♂). In each cross-group, 20 newly emerged females and 20 newly emerged males were transferred to each cage and reared as mentioned above. Briefly, 5-day-old females were offered a blood meal and the eggs subsequently harvested. Post hatching, the number of viable larvae from each cross was used to determine the level of *w*AlbB-induced CI. The number of hatched eggs was counted under the dissecting microscope and recorded. CI was statistically analysed under the following hypothesis: at least one pair of all groups is insignificant as compared to the average percentage egg hatch against the alternative that there is at least one difference.

#### Mating competitiveness

The male competitiveness index (C) calculation was adopted from Zhang et al. [43]. Briefly, the mating competitiveness trial of WAG involved four WAG:RAG male ratios (0:40, 20:20, 30:10, 40:0). The 40 virgin (WAG/RAG) males (72–96 h post emergence) followed by 30 virgin RAG females (48–72 h post emergence) were released into field cages. ANOVA was used to compare the groups under the following hypotheses. (i) H_11_, at least in one group the number of laid eggs is different; (ii) in H_12_, at least in one group he hatch proportion is different.

#### Life span (with and without food)

The longevity of 25 virgin WAG F_8_ (*Wolbachia*-infected) adults (females and males) was estimated while maintained on 10% glucose only or without any food. Triplicates of RAG adults were used as control. Larvae could pupate as described above, and all the male and female pupae were manually separated based on body size. To ensure virginity, the pupae were then transferred to an individual test tube (13 × 100 mm; Fisher Scientific Company LLC , Pittsburgh, PA, USA) containing 40 ml of distilled deionized water. Pupae remained in test tubes until adult emergence. Twenty-five mosquitoes, either males or females, were transferred to round paper cups (volume: 946.4 ml; model H4325-J8000, Symphony®; Dart Container, Mason, MI, USA) having a white fine fabric net on the top. Dead mosquitoes were recorded and removed from the opening at the bottom of the container every day until no viable mosquitoes were left.

### Overview of data analysis

The data of all the experiments were analysed using the appropriate parametric and non-parametric statistical tests, such as the Chi-square test of association and ANOVA test for parametric data, and the Wilcoxon–Mann–Whitney test and Mantel–Cox test for non-parametric data. Data on fecundity, mating competitiveness and CI assays were tested using ANOVA at a 95% CI, using the SPSS software package (SPSS IBM Corp., Armonk, NY, USA). The details of each test have been mentioned above with the corresponding experimental design.

## Results

### *Wolbachia* transinfection via microinjection

Four experimental groups of RAG eggs (total* n* = 376) received cytoplasm via microinjection from the donor *Ae. albopictus* carrying *w*AlbA and *w*AlbB *Wolbachia* strains. Only 44 of the inoculated WAG eggs hatched, with 25 neonate larvae surviving up to the second instar. From these 25 larvae, 20 adults ultimately emerged, nine of which were morphologically identified as female and the remaining 11 as male (Additional file 1: Table S1); these adults were denoted the F_0_ generation. None of the eggs hatched in the second experiment and, therefore, this group was discarded. All nine WAG virgin females were outcrossed with RAG males (uninfected *Ae. aegypti*); thus, F_1_ WAG eggs were obtained from each F_0_ female separately for 2 days. All F_0_ adults were then screened by PCR targeting the *wsp* gene using *w*AlbA and *w*AlbB primers separately, as described in the Methods section.

In total, 13 F_0_ WAG (7 females, 6 males) mosquitoes were found to be positive for *Wolbachia* infection (Additional file 1: Table S2). A gel image of the PCR products using strain-specific *wsp* gene primers is shown in Additional file 1: Figure S3. The remaining uninfected F_0_ 7 adults were discarded along with their eggs. In addition, the males were not used in subsequent steps to establish the *Wolbachia* infected line and, therefore, a gel image of infection status in males is not shown.

### Double infection in WAG in the F_0_ and F_1_ generations

The WAG F_0_ females were found to be infected with *w*AlbA and/or *w*AlbB *Wolbachia* in all three possible combinations. One, four and two females were harboured a single *w*AlbA, double *w*AlbA + *w*AlbB and single *w*AlbB infection, respectively. Screening showed that four and two F_0_ males were double infected (*w*AlbA + *w*AlbB) and *w*AlbB single infected, respectively (Additional file 1: Table S3).

A total of 334 F_1_ WAG eggs were harvested from the five F_0_ females. Of these, 132 eggs hatched, with 115 larvae surviving to become F_1_ adults (Additional file 1: Table S4). In total, 17 F_1_ females were *Wolbachia*-infected (Additional file 1: Table S5), of which five, two and 10 females were infected with *w*AlbA, *w*AlbA + *w*AlbB and *w*AlbB, respectively (Additional file 1: Table S6). Two females carrying double *Wolbachia* strains were selected to establish the WAG line.

### *Wolbachia* infection in WAG at the F_5_ generation

Randomly selected  12 virgin females and 12 males from the WAG F_5_ stock line were screened for double infection of *Wolbachia* strains using the PCR assay. All individuals were found infected by *w*AlbB only. Not a single female (Additional file 1: Figure S4) or male was infected with *Wolbachia w*AlbA single infection or with *w*AlbA + *w*AlbB double infection, possibly due to the low infection rate of the *w*AlbA strain. Subsequently, randomly selected individuals from stock cages were screened at various generations, and *Wolbachia* infection was consistently confirmed up to the F_85_ generation.

### Egg hatching rate in WAG up to the F_8_ generation

An overview of the egg hatching rate of WAG mosquitoes over eight generations after *Wolbachia* transfection is shown in Additional file 1: Figure S5. In the first three generations, the fertility of WAG was low, ranging from 45 to 29%. However, a 70% egg hatch was achieved in the F_5_ generation, and after the F_6_ generation the fertility of WAG was observed to be stable at 80 ± 5%.

### Weather conditions during the semi-field experiments

Average daily temperatures and relative humidity during the 3 months of the semi-field trials ranged from 18 °C to 31 °C, and from 54% to 92%, respectively. Total rainfall was noted as 442.2 mm in > 15 episodes, resulting in suitable weather conditions for the mosquito population (Additional file 1: Figure S6).

### General fitness of WAG in the semi-field experiments

#### Female fecundity and fertility

**Fig. 1:**
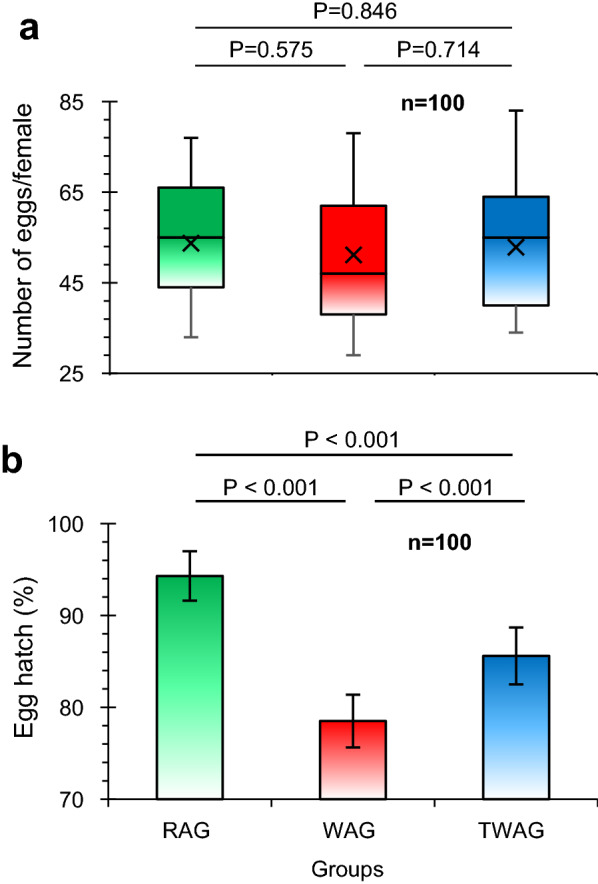
Impact of *w*AlbB *Wolbachia* on oviposition (**a**) and egg hatching rate (**b**) of WAG females. Box-and-whisker plot (**a**) represents the number of eggs laid per female in the respective *Wolbachia*-infected and non-infected groups. The bottom and top of the box represent the first (lower) and third (upper) quartiles (25–75th percentile), respectively, and the middle line within the box indicates the median. The cross denotes the mean. The vertical lines (ends of the whiskers) extend to the minimum and maximum values. *P*-values obtained by ANOVA at 95% confidence interval to compare the difference of means of the three groups are mentioned at the top of **a** and *P*-values obtained using a Chi-square test to compare the three groups are given at the top of **b**. Abbreviations: ANOVA, Analysis of variance; RAG, uninfected *Aedes aegypti* (control); TWAG, *w*AlbB *Wolbachia* removed by tetracycline; WAG, *w*AlbB *Wolbachia*-transfected colony treatment.

Fecundity is a measure of the reproductive potential of female mosquitoes. There was no significant difference (*P* > 0.05) in egg-laying capacity (mean: 52.6 per female) between the three groups (Fig. 1a). Egg hatching rates of the RAG, WAG and TWAG groups were 94.3, 78.5 and 85.6%, respectively. The Chi-square test of association indicated that egg hatching rates among all the three groups were significantly different (*P* < 0.001) (Fig. 1b). This result demonstrated that there was no considerable effect of *w*AlbB on fecundity whereas a remarkable decrease in fertility was noted with *Wolbachia* infection.

#### Larval development and wing-length measurement

**Fig. 2:**
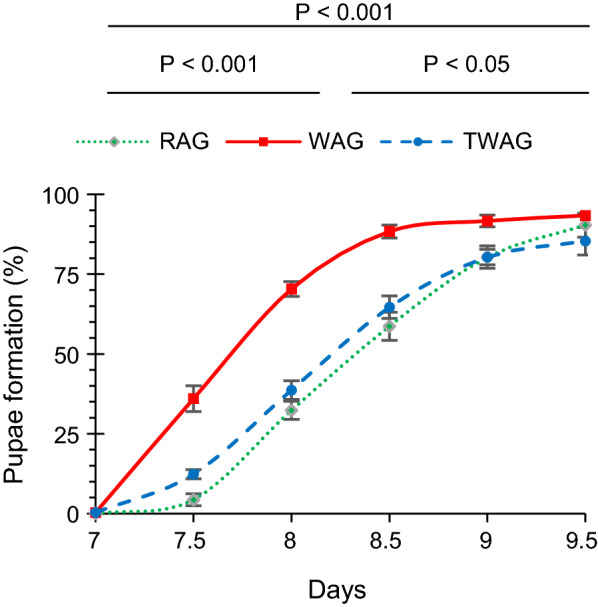
Effect of *w*AlbB on the pupae formation of WAG. Day number represents time post egg hatching. Error bars represent the SEM of three biological replicates. *P*-values obtained by the test of association between days and pupae are given between the three groups on the top of the legends. Abbreviations: SEM, Standard error of the mean; *w*AlbB, *Wolbachia* strain B that naturally infects *Aedes* *albopictus*

Figure 2 shows that pupal emergence was significantly higher (*P* < 0.001) at day 7.5 in the WAG group than in the RAG group (36% vs. 4%, respectively). In addition, at day 8.5, pupal emergence was 88% in WAG and 59% RAG. However, a 93% pupal emergence was recorded in WAG as compared to 90% in RAG at day 9.5. Based on these observations, it could be inferred that *w*AlbB induced the earlier development of larvae to pupal formation in *Ae. aegypti*.

**Fig. 3:**
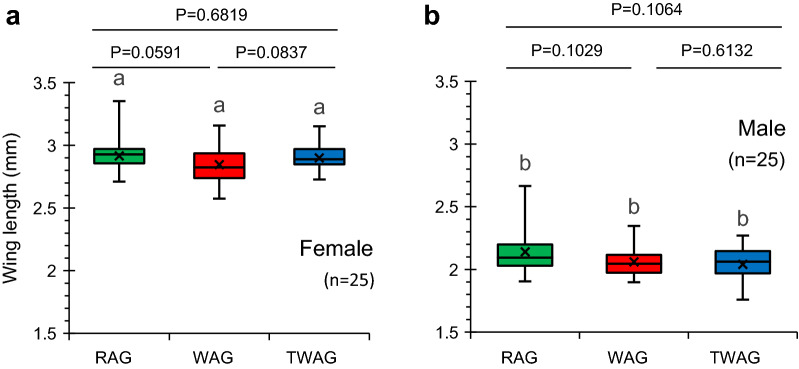
Impact of *Wolbachia w*AlbB strain on wing size of *Ae. aegypti* females (**a**) and males (**b**) at the F_8_ generation. Similar lowercase letters above each box indicate that these is no significant difference (*P* > 0.05) between the groups by Wilcoxon–Mann–Whitney test at the 95% confidence interval of the difference. *P*-values are given at the top of each graph. The box-and-whisker plots represent the observed distribution of wing length in *Wolbachia-*infected and non-infected groups. The bottom and top of the box represent the first (lower) and third (upper) quartiles (25–75th percentile), respectively, and the middle line within each box indicates the median. The cross represents the mean. The vertical lines (ends of the whiskers) extend to the minimum and maximum values

Analysis of the data sets on wing lengths of RAG, WAG and TAG females (range: 2.57–3.16 mm) and males (range: 1.57–2.92 mm) indicated no significant between-group difference (*P* > 0.05) using the Mann–Whitney U-test (Fig. 3). Therefore, we concluded that infection with *w*AlbB did not affect the body size of the host, based on wing length in either sex.

#### Cytoplasmic incompatibility

**Fig. 4:**
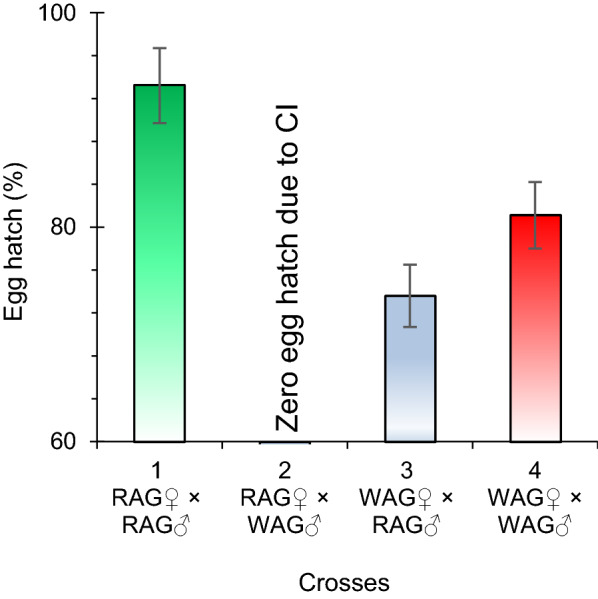
Percentage egg hatch rate in various possible cross combinations between females (♀) and males RAG (♂) of the RAG and WAG F_9_ generation for CI evaluation. Error bars represent the SEM of three biological replicates. The eggs did not hatch in experimental group 2 due to CI, where wild RAG females were crossed with WAG (*w*AlbB *Wolbachia* infected) males. Abbreviations: CI, Cytoplasmic incompatibility

The potential of *w*AlbB to induce CI was determined by allowing WAG mosquitoes to cross with RAG mosquitoes. A maximum egg hatch of 93.2% was noted in a cross between RAG female mosquitoes (RAG♀) × RAG male mosquitoes (RAG♂), while 0% egg hatch was observed in the cross between RAG♀ × WAG♂. Therefore, complete (100%) CI was induced. An average of 73.6% egg hatch was observed in the WAG♀ × RAG♂ group. In addition, the egg hatch was 81.1% in a group involving WAG♀ × WAG♂ (Fig. 4).

These results indicated that the *w*AlbB *Wolbachia* strain induced complete CI in *Ae. aegypti* when *Wolbachia*-infected males were crossed with uninfected females (*P* > 0.001). The least significant difference test (LSD) was applied, and results indicated that one pair was not significant among the six pairs tested.

#### Mating competitiveness assays

**Fig. 5:**
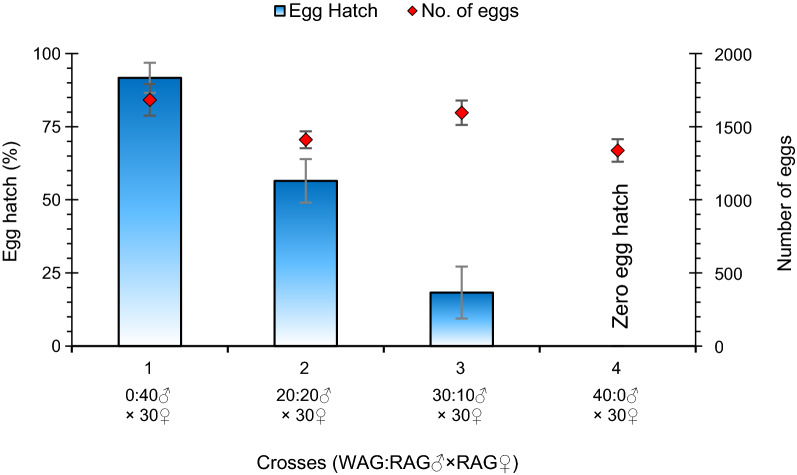
Egg hatch rate and the number of eggs laid by wild RAG females as a result of crosses 2 at various WAG:RAG male ratios. Error bars represent the SEM of three biological replicates. Group 4 indicates complete population suppression due to CI when *w*AlbB *Wolbachia*-infected WAG males of the F_8_ generation mate with wild RAG females

Thirty female and 40 male mosquitoes were placed together in the same cage for 2 days. A maximum of 1684 eggs was collected from the control group (RAG♂ × RAG♀). No remarkable difference in egg-laying capacity was noted in all four groups. However, the number of eggs that eventually hatched was significantly different ( P < 0.001), indicating that the number of compatible matings was different in each group. The egg hatch in the compatible cross (RAG♂ × RAG♀) was 91.7%. At a ratio of 20:20 WAG:RAG males, egg hatching was 56.5%, giving a competitiveness index of 0.63. This means that compatible (RAG) males were slightly more competitive than incompatible (WAG) males (Table 1). A 35.2% reduction in egg hatch was noted when WAG males mated with RAG females when in competition with RAG males. At a 3:1 WAG:RAG release ratio, egg hatching was reduced to 18.3% due to maximum matings of incompatible males (WAG♂) (Fig. 5). Thus, egg hatch was significantly influenced by the ratio of WAG males.

**Table 1:** Competitiveness index of different ratios of F_8_ WAG males measured at different ratios of RAG males in semi-field conditions

Male ratio WAG:RAG	♂ × ♀ WAG:RAG × RAG	Egg hatch^a^ (*n* eggs)	*Hc*-*Hr*	*Hr*-*Hi*	*Cm*/*In*	Competitiveness index^b^
0:1	0:40 × 30 *Hc*	91.7% ± 5.1^a^ (1684)	Negative control group			
1:1	20:20 × 30 *Hr*	56.5% ± 8.1^b^ (1411)	35.2 ± 11.8	56.5 ± 8.1	1	0.63 ± 0.3
3:1	30:10 × 30 *Hr*	18.3% ± 5.9^c^ (1595)	73.4 ± 11.0	18.3 ± 5.9	0.33	1.38 ± 0.6
1:0	40:0 × 30 *Hi*	0.0% ± 0.0^d^ (1338)	Positive control group			

All values are given as ± standard error of the mean of triplicate measures

*RAG* Uninfected *Aedes aegypti* (control),* TWAG* *w*AlbB *Wolbachia* removed by tetracycline, *WAG* *w*AlbB *Wolbachia*-transfected colony treatment

^a^Different lowercase letters indicate that the values are statistically different (*P* < 0.05) in all crosses using Tukey mean procedure test

^b^$\mathrm{Competitiveness index}=\frac{Hc-Hr}{Hr-Hi }\times \frac{Cm}{In}$, where *Hc* = hatch rate of eggs harvested from the cross RAG♂ × RAG♀ (compatible); *Hr* = hatch rate of eggs harvested from the cross WAG:RAG♂ × RAG♀; *Hi* = hatch rate of eggs harvested from the cross WAG♂ × RAG♀ (incompatible); *Cn* = number of compatible males (RAG); *In* = number of incompatible males (WAG)

#### Life span (with and without food)

**Fig. 6:**
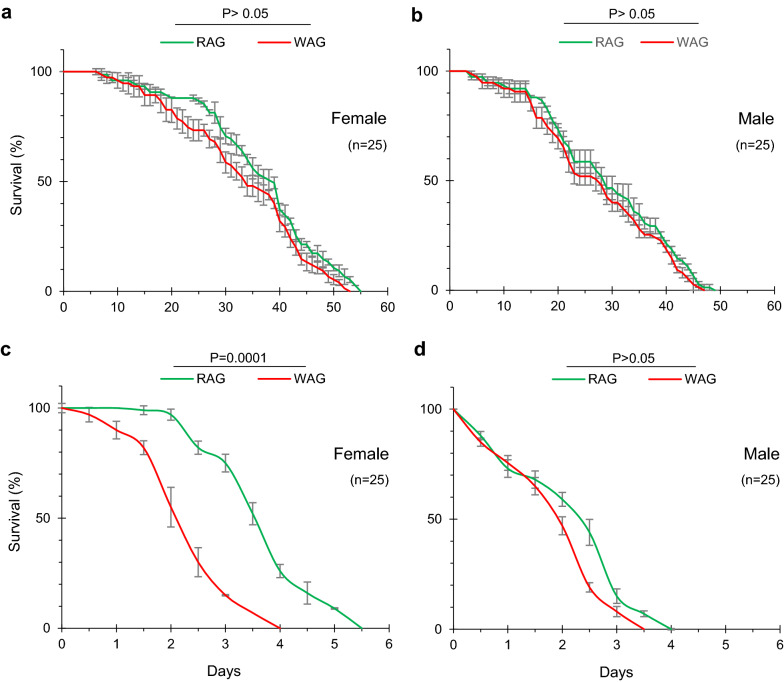
Impact of *w*AlbB *Wolbachia* on the life span of adult WAG F_8_ females (**a**) and males (**b**) with food, and in females (**c**) and males (**d**) without food. Day number represents age starting from adult emergence. Error bars represent the SEM of three biological replicates. Data were analysed using the Mantel-Cox test

Longevity was not significantly different in RAG and WAG females (Mantel–Cox test, *P* > 0.05). Maximum survival of RAG and WAG females was 54 and 52 days, respectively. The survival curve was almost similar between the two groups up to 14 days (at 93% survival). However, a noticeable decrease in survival in WAG females was observed from 15 days (89% survival) up to 26 days (73% survival). After 40 days, the death rate was similar in both groups (Fig. 6a). Similarly, RAG and WAG males showed similar survival patterns (Mantel–Cox test, *P* > 0.05), with an initial survival stability of 2 weeks. The survival curve was notably similar ,with > 90% of male mosquitoes alive up to 15 days. At 28 days, 51–55% of males were still alive in both groups. A maximum survival of 49 and 47 days was observed in the male RAG and WAG groups, respectively (Fig. 6b).

Under conditions of complete starvation, WAG females had a significantly shorter life span, with a maximum survival of 4 days, than RAG females, with a maximum survival of > 5 days (Mantel–Cox test, *P* < 0.0001). After 1.5 days, the number of WAG females began to gradually decrease (Fig. 6c). Life span assays indicated no significant difference in the survival curve of RAG and WAG males under conditions of complete starvation (*P* > 0.05) (Fig. 6d). Males of both groups had a maximum life span of 4 days. *w*AlbB remarkably reduced the survival of WAG *Ae. aegypti* females compared with RAG females under starvation conditions, while *w*AlbB did not affect the survival rate of male *Ae. aegypti* under the same conditions*.*

## Discussion

The results of the current study suggest that embryonic microinjection is a suitable strategy for the interspecific transfer of *Wolbachia*. *Wolbachia* affects various phenotypes of the mosquitoes in which it is present, such as reductions in life span and fecundity, respectively [44–48]. In our study, the *Wolbachia* donor (*Ae. albopictus*) and recipient (*Ae. aegypti*) mosquitoes were locally collected from Lahore, Pakistan. It was expected that these indigenous mosquito species that have a local mitochondrial haplotype would be better adapted to the warm climate of the country than nonlocal species and that these *Wolbachia*-infected mosquitoes would have high chances of survival and progression in the natural weather conditions following field releases. These mosquitoes would thus have a high chance of mating with the females of the wild population. Similarly, the local *Wolbachia* strain would show good CI induction, virus inhibition and fitness cost on the host, among other effects.

In the present study, *w*AlbB *Wolbachia* was successfully established in naturally uninfected *Ae. aegypti* from wild-collected *w*AlbA + *w*AlbB double-infected *Ae. albopictus*. The recipient *Ae. aegypti* colony was initially double infected at the F_0_ generation and then later the double infection was replaced by a single infection of *w*AlbB *Wolbachia* within five generations post microinjection. The exact number of filial (F_1_–F_5_) generations needed for the loss of *w*AlbA infection to occur or the reason behind this removal was not assessed. However, it is important to mention here that to eliminate genetic bottlenecks, we performed outcrosses of double-infected (*w*AlbA + *w*AlbB) F_0_ females with uninfected males. One of the possible reasons for the loss of *Wolbachia* is the low titre of the *w*AlbA *Wolbachia* in the F_0_ females. The *w*AlbA might be removed simultaneously or gradually during these outcrosses. Afterwards, the *w*AlbB infection is currently stable in successive generations (up to F_85_ and thereafter). Xi et al. [29] reported the transfer of embryonic cytoplasm from double-infected *Ae. albopictus* to *Ae. aegypti*. The *w*AlbA infection was unstable, and only the *w*AlbB strain of *Wolbachia* was established successfully in *Ae. aegypti* (WB1)*.* Similarly, the cytoplasm of double-infected *Ae. albopictus* was microinjected separately into the aposymbiotic *Wolbachia*-removed *Ae. albopictus* (Houston) as well as the *Ae. aegypti* (WB2). A single stable *w*AlbB infection was established in the host [29, 49]. In contrast, the *w*AlbA strain was not always lost after transinfection [50, 51].

Many studies have reported that the *w*AlbB strain of *Wolbachia* has the potential to be used as a biocontrol agent for the control of different diseased vectors [29, 30, 36, 41, 47, 52–54]. The current study was also focused on evaluation of the effect of *w*AlbB on the general fitness of the host, such as fecundity, wing length, life span assays and, most importantly, the CI of *Ae. aegypti* under semi-field conditions*.* The field conditions are highly variable in any part of the world as compared to standard laboratory conditions. Factors such as temperature, humidity, wind, rainfall and the day/night cycle greatly affect the efficiency or even survival of laboratory-reared mosquitoes. Experiments under semi-field conditions therefore provide more reliable data for predicting the results of field trials.

To assess the changes in the physiology of the host, natural or artificial *Wolbachia* infection can be removed from insects by treatment with various antibiotics, including tetracycline [55, 56] and rifampicin [57]. The results of the present study suggest that *Wolbachia* did not affect the physiology of *Ae. aegypti*, as indicated by the fecundity and wing length measurements. Similarly, Calviti et al. [58] reported that the removal of *Wolbachia* infection had no observable effect on the fitness of the natural host *Ae. albopictus* under either laboratory conditions or in greenhouses.

In the current study, *Wolbachia* strain *w*AlbB had no impact on the fecundity of *Wolbachia*-infected females in the semi-field experiments, which is consistent with the results from previous laboratory studies [59, 60]. However, a significant decrease in the egg hatching rate was noted, which is also consistent with results of previous studies [25, 29, 61]. On the other hand, different authors [41, 62] have suggested that *w*Mel-infected *Ae. aegypti* and *w*AlbB-infected *Anopheles stephensi* females showed reduced fecundity compared to uninfected mosquitoes at high temperature.

Wing-length measurements have been used in mosquito studies to infer the overall body size, which in turn is a measure of general fitness, including the mating potential of the mosquitoes [42, 63, 64]. In the current study, *w*AlbB *Wolbachia* did not have any negative impact on the wing size of the host. These results are consistent with the findings of Axford et al. [65]. No significant impact of *w*AlbB *Wolbachia* was reported on the body size of *Ae. aegypti*. Furthermore, current results are also consistent with previous reports of *w*AlbB and *w*Mel not having any significant impact on the wing length/body size of *Ae. aegypti* [62] and *An. stephensi* [41], respectively.

By releasing different ratios of RAG and WAG males to RAG females, we found that *Wolbachia*-infected males were competitive with wild males. These findings are consistent with those of a previous study [66]. Conversely, Xi et al. [67] reported that *Wolbachia* reduced the mating competitiveness of transfected male mosquitoes.

It is well documented that the same strain of *Wolbachia* not only imparts a different impact on the host of another species but also on the host of the same species. In search of the *Wolbachia* strain for better features, different insects have been screened. It is also evident from the results that the selected *w*AlbB strain affected the host differently. The current results are broadly consistent with previously published data, with minor differences regarding egg hatching, larval development and life span that could be due to genetic differences in background or density of the *w*AlbB strain. It is important to mention that *Wolbachia*-infected mosquitoes have been released in the field in Australia [2] and China [68]. Moreover, *Wolbachia* strain *w*AlbB has been documented to reduce dengue transmission in Malaysian populations of *Ae. aegypti* in field trials [36].

## Conclusions

In the present study, *Wolbachia* strain *w*AlbB produced complete CI by affecting fertility in the new host *Ae. aegypti* and reduced the life span of only females under starvation conditions in the semi-field experiments. *Wolbachia* strain *w*AlbB did not affect the fecundity of female mosquitoes but significantly decreased the rate of egg hatching. This *w*AlbB strain has a great potential to control the dengue vector *Ae. aegypti* population by producing 100% CI without affecting the general fitness of the host under natural conditions. As such, this strain could be used as biocontrol for vector-borne diseases.

## Supplementary Information

**Additional file 1: Fig. S1.** General (**a**) and strain-specific primer sequences (**b**, **c**) showing estimated product size along with thermal cycler conditions for the detection of *Wolbachia* by targeting the *wsp* gene. **Fig. S2.** A semi-field cage was used for the evaluation of the fitness of WAG. **Fig. S3.** Gel electrophoresis analysis of PCR products using *wsp* gene-based strain-specific *w*AlbA (**a**) and *w*AlbB (**b**) primers targeting gDNA of WAG F_0_ females. **Fig. S4.** Gel electrophoresis analysis of PCR products using *Wolbachia* wAlbA-specific (**a**, **c**) and wAlbB-specific (**b**, **d**) primers targeting the *wsp* gene from WAG F_5_ females (**a**, **b**) and males (**c**, **d**). **Fig. S5.** Egg hatching rate of WAG females from F_1_ to F_8_ generation. **Fig. S6.** Weather conditions from August to October 2016 during semi-field experiments: mean daily temperature (**a**), relative humidity (**b**) and rainfall (**c**). **Table S1.** Survival details of *Ae. aegypti* embryos (F_0_) post microinjection of cytoplasm from *Ae. albopictus* embryos. **Table S2.** *Wolbachia* infection along with gender distribution in WAG F_0_ adults post microinjection. **Table S3.** Distribution of double infection of *Wolbachia* strains in parental (F_0_) WAG adults post microinjection. **Table S4.** Egg hatching rate of WAG F_1_ eggs. **Table S5.** Survival and *Wolbachia* infection details of WAG F_1_. **Table S6.** Details of *Wolbachia* positive WAG F_1_ females post microinjection.

## Data Availability

Further details of the current study are available from the corresponding author on reasonable request.

## Abbreviations

**♀**: Female(s)
**♂**: Male(s)
ANOVA: Analysis of variance
CI: Cytoplasmic incompatibility
F: Filial
RAG: *Aedes aegypti* lacking *Wolbachia* naturally
SEM: Standard error of the mean
TWAG: *w*AlbB infection removed in WAG by antibiotic treatment
WAG: *w*AlbB-infected *Ae. aegypti*
*Wsp*: *Wolbachia* outer surface protein
