# Supplementary material for: Establishment of Wolbachia infection in Aedes aegypti from Pakistan via embryonic microinjection and semi-field evaluation of general fitness of resultant mosquito population
Source: Parasit Vectors. 2022 Jun 6;15:191. doi: 10.1186/s13071-022-05317-4 (PMC9169386; doi:10.1186/s13071-022-05317-4)
Supplement: Supplementary file 1 — Additional file 1: Fig. S1. General (a) and strain-specific primer sequences (b, c) showing estimated product size along with thermal cycler conditions for the detection of Wolbachia by targeting the wsp gene. Fig. S2. A semi-field cage was used for the evaluation of the fitness of WAG. Fig. S3. Gel electrophoresis analysis of PCR products using wsp gene-based strain-specific wAlbA (a) and wAlbB (b) primers targeting gDNA of WAG F0 females. Fig. S4. Gel electrophoresis analysis of PCR products using Wolbachia wAlbA-specific (a, c) and wAlbB-specific (b, d) primers targeting the wsp gene from WAG F5 females (a, b) and males (c, d). Fig. S5. Egg hatching rate of WAG females from F1 to F8 generation. Fig. S6. Weather conditions from August to October 2016 during semi-field experiments: mean daily temperature (a), relative humidity (b) and rainfall (c). Table S1. Survival details of Ae. aegypti embryos (F0) post microinjection of cytoplasm from Ae. albopictus embryos. Table S2. Wolbachia infection along with gender distribution in WAG F0 adults post microinjection. Table S3. Distribution of double infection of Wolbachia strains in parental (F0) WAG adults post microinjection. Table S4. Egg hatching rate of WAG F1 eggs. Table S5. Survival and Wolbachia infection details of WAG F1. Table S6. Details of Wolbachia positive WAG F1 females post microinjection. [file 13071_2022_5317_MOESM1_ESM.pdf]

Additional file 1.

Sr.	**Primer sequences** 5'-3'<br>**Estimated Product size** (Start-End)	Units	Denaturation		Annealing	Extension		Storage
**a**	*wsp*_F<br>AAGGAACCGAAGTTCATG<br>18 bases<br>*wsp*_ R<br>AAAAATTAAACGCTACTCCA<br>20 bases<br>**501 bp** (183-691)				30 Cycles			
		**(°C)**	**94.0**	**95.0**	**55.0**	**72.0**	**72.0**	**4.0**
		(Min)	5:00	0:05	0:30	0:30	5:00	α
**b**	*w*AlbA_*wsp*_qRT_F<br>GTGTTGGTGCAGCGTATGTC<br>20 bases<br>*w*AlbA_*wsp*_qRT_R<br>GCACCAGTAGTTTCGCTATC<br>20 bases<br>**187 bp** (330-516)				37 Cycles			
		**(°C)**	**98.0**	**98.0**	**56.0**	**72.0**	**72.0**	**4.0**
		(Min)	6:00	0:05	0:30	0:45	5:00	α
**c**	*w*AlbB_*wsp*_qRT_F<br>ACGTTGGTGGTGCAACATTTG<br>21 bases<br>*w*AlbB_*wsp*_qRT_R<br>TAACGAGCACCAGCATAAAGC<br>21 bases<br>**268 bp** (106-373)				35 Cycles			
		**(°C)**	**94.0**	**95.0**	**55.0**	**72.0**	**72.0**	**4.0**
		(Min)	5:00	0:05	0:30	0:30	5:00	α

Fig. S1 General (**a**) and strain-specific primer sequences (**b, c**) showing estimated product size along with thermal cycler conditions for the detection of *Wolbachia* by targeting the *wsp* gene.

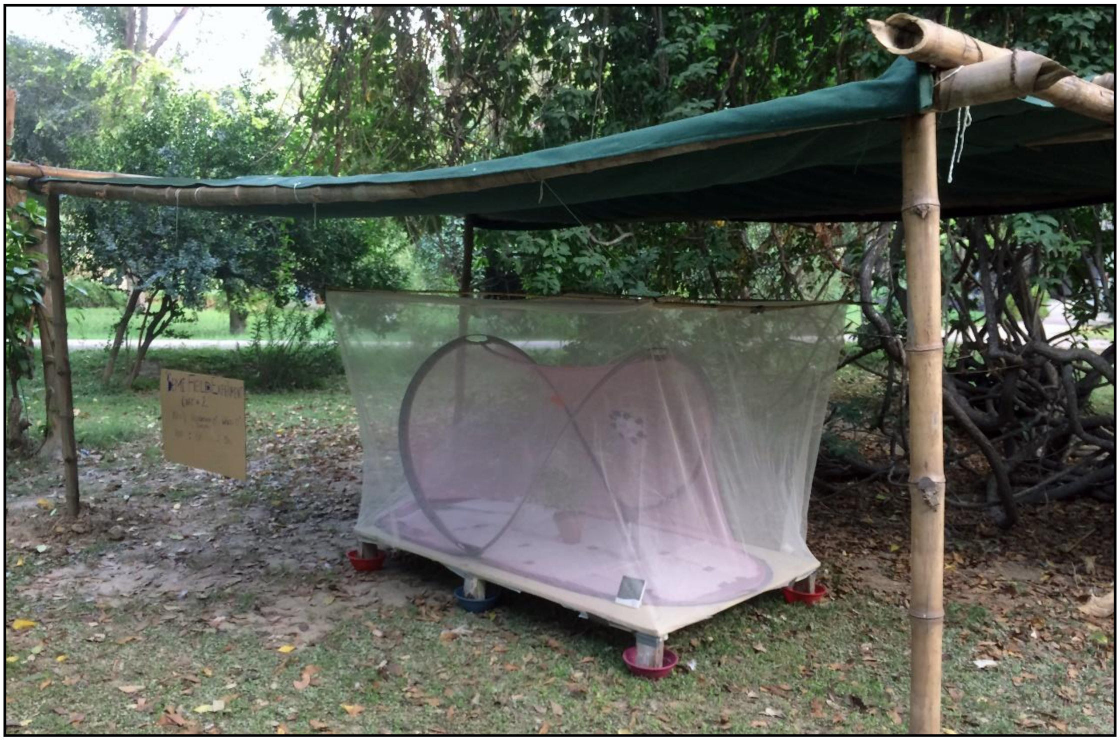

Fig. S2 A semi-field cage used for the evaluation of the fitness of WAG.

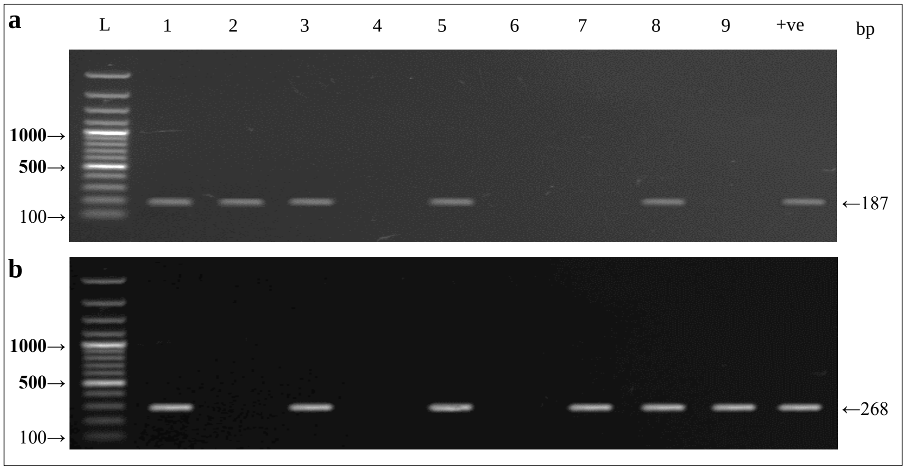

Fig. S3 Gel electrophoresis analysis of PCR products using *wsp* gene-based strain-specific *w*AlbA (**a**) and *w*AlbB (**b**) primers targeting gDNA of WAG F_0 females.

L, Ladder 1kb; **1-9**, samples; **+ve,** *w*AlbA (A) and *w*AlbB (B) infected *Ae. albopictus*; **bp**, base pair.

Sample number **1** from experimental group 1, **2-7** from experimental group 3 and **8-9** from experimental group 4.

2% agarose gel in 1× TAE under 120 volts for 30 min.

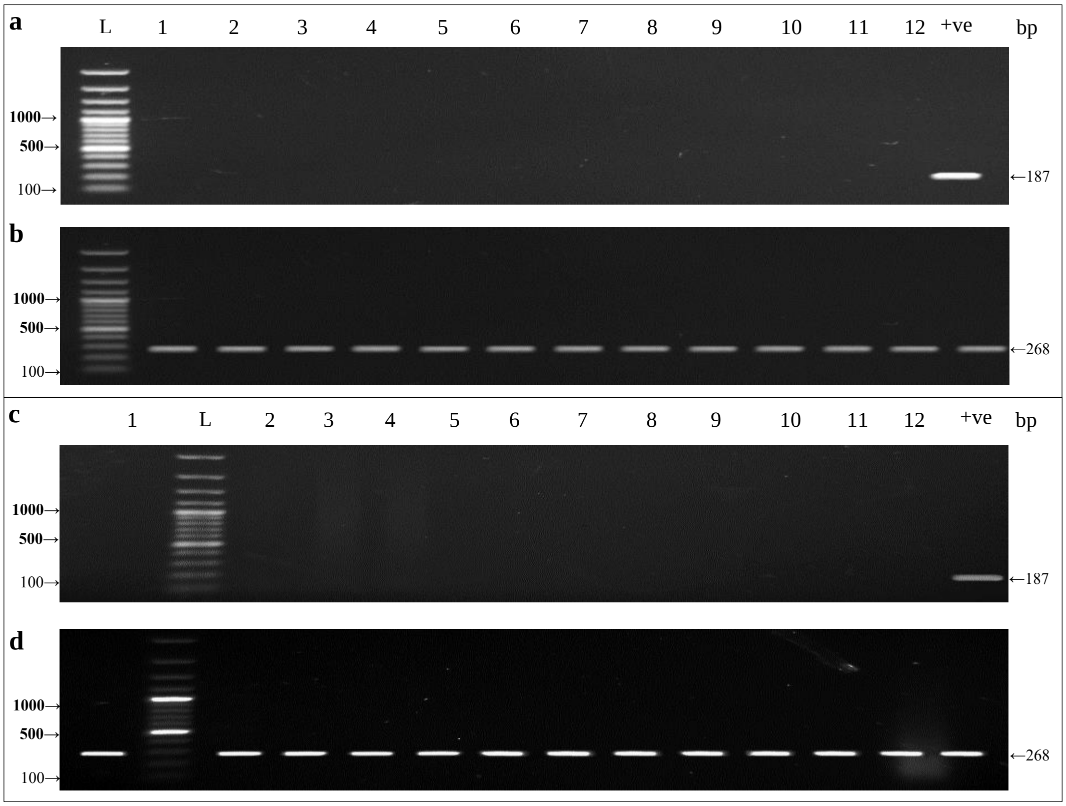

Fig. S4 Gel electrophoresis analysis of PCR products using *Wolbachia* wAlbA (**a** & **c**) and wAlbB (**b** & **d**) strains specific primers targeting the *wsp* gene from WAG F_5 females (**a-b**) and males (**c-d**).

L, Ladder 1 kb; **1-12**, samples; **+ve**, *w*AlbA/*w*AlbB infected *Ae. albopictus*; **bp**, base pair.

2% agarose gel in 1× TAE under 120 volts for 30 min.

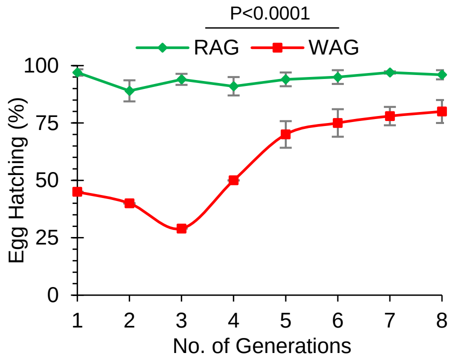

Fig. S5 Egg hatching rate of WAG females from F_1 to F_8.

RAG, Uninfected *Ae. aegypti* (control); **WAG**, *w*AlbB *Wolbachia* transfected colony.

P-values obtained by the test of association between the groups are mentioned above in the legends.

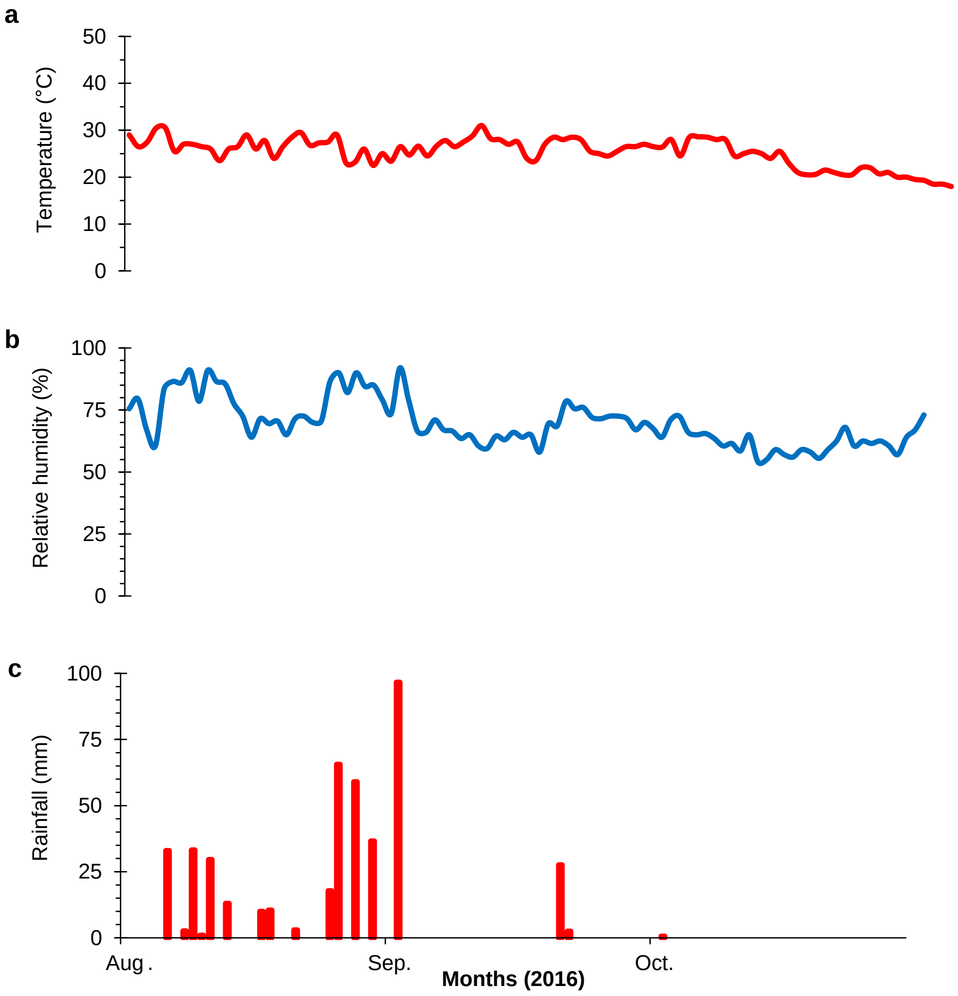

Fig. S6 Weather conditions from August to October 2016 during semi-field experiments Mean daily temperature **(a)**, relative humidity (**b)** and rainfall **(c)**.

Table S1 Survival details of *Ae. aegypti* embryos (F_0) post microinjection of cytoplasm from *Ae. albopictus* embryos.

**Experimental Group**	**Hatch rate** (Hatch/ Injected eggs)	**Larvae** (2nd Instars/Hatched)	**Pupae** (Pupae/2nd Instars)	**Adults** (Adult/Pupae)	**Female sex ratio** (Female/Total)
1	15.23% (32/210)	43.75% (14/32)	71.42% (10/14)	100% (10/10)	10.00% (1/10)
2*	0.00% (0/50)	Group discarded due to zero hatch rate.			
3	16.67% (10/60)	90.00% (9/10)	88.89% (8/9)	100% (8/8)	75.00% (6/8)
4	3.57% (2/56)	100% (2/2)	100% (2/2)	100% (2/2)	100% (2/2)
**Total**	**11.70%** (44/376)	**56.82%** (25/44)	**80.00%** (20/25)	**100%** (20/20)	**45.00%** (9/20)

Table S2 *Wolbachia* infection along with gender distribution in WAG F_0 adults post microinjection.

**Experimental Group**	**F_0** (Female:Male)	***Wsp* Positive F_0**		
		**Total**	**Female** (Positive/Total)	**Male** (Positive/Total)
1	10 (1:9)	60.0% (6/10)	100% (1/1)	55.6% (5/9)
2	Group discarded due to zero hatch rate.			
3	8 (6:2)	62.5% (5/8)	66.7% (4/6)	50.0% (1/2)
4	2 (2:0)	100% (2/2)	100% (2/2)	-
**Total**	**20** **(9:11)**	**65.0%** **(13/20)**	**77.8%** (7/9)	**54.5%** (6/11)

WAG, *Wolbachia* transfected *Ae. aegypti*; *Wsp*, *Wolbachia* surface protein gene

Table S3 Distribution of double infection of *Wolbachia* strains in parental (F_0) WAG adults post microinjection.

Experimental Group	Female/ Male	*Wolbachia* positive F_0		
		***w*AlbA**	***w*AlbA + *w*AlbB**	***w*AlbB**
1	1	-	1	-
	5	-	4	1
2	Group discarded due to zero hatch rate.			
3	4	1	2	1
	1	-	-	1
4	2	-	1	1
	-	-	-	-
**Total**	**7**	**1**	**4**	**2**
	**6**	**-**	**4**	**2**

WAG, *Wolbachia* transfected *Ae. aegypti*

Table S4 Egg hatching rate of WAG F_1 eggs.

**Experimental Group**	**Female ID**	***Wolbachia* Strain**	**No. of eggs laid**	**Hatch rate** (Hatch/ Total)
1	1.1	*w*AlbA + *w*AlbB	No eggs	-
3	3.2	*w*AlbA only	No eggs	-
	3.3	*w*AlbA + *w*AlbB	73	31.51% (23/73)
	3.4	*w*AlbA + *w*AlbB	48	14.58% (7/48)
	3.5	*w*AlbB only	85	43.53% (37/85)
4	4.6	*w*AlbA + *w*AlbB	89	73.03% (65/89)
	4.7	*w*AlbB only	39	0.00% (0/39)
**Total**	**7**		**334**	**39.52%** (132/334)

WAG, *Wolbachia* transfected *Ae. aegypti*

Table S5 Survival and *Wolbachia* infection details of WAG F_1.

**Experimental Group**	**Female identity number**	***Wolbachia* strain**	**1st Instar**	**Adults** (Adult/1st Instar)	**Female sex ratio** (Female/Total)
1	1.1	*w*AlbA + *w*AlbB	-	-	-
3	3.2	*w*AlbA only	-	-	-
	3.3	*w*AlbA + *w*AlbB	23	82.6% (19/23)	47.4% (9/19)
	3.4	*w*AlbA + *w*AlbB	7	85.7% (6/7)	66.7% (4/6)
	3.5	*w*AlbB only	37	89.2% (33/37)	27.3% (9/33)
4	4.6	*w*AlbA + *w*AlbB	65	87.7% (57/65)	38.6% (22/57)
	4.7	*w*AlbB only	-	-	-
**Total**			**132**	**87.1%** (115/132)	**43.8%** (44/115)

WAG, *w*AlbB *Wolbachia* transfected *Ae. aegypti*

Table S6 Details of *Wolbachia* positive WAG F_1 females post microinjection.

Experimental group	Female identity number	*Wolbachia* infected F_1 (+ve/Total)	*Wolbachia* strains		
			*w*AlbA	*w*AlbA + *w*AlbB	*w*AlbB
1	1.1	-	-	-	-
3	3.2	-	-	-	-
	3.3	11.11% (1/8)	-	1	-
	3.4	50.00% (2/4)	1	-	1
	3.5	22.22% (2/8)	-	-	2
4	4.6	54.54% (12/22)	4	1	7
	4.7	-	-	-	-
Total	7	40.48% (17/42)	5	2*	10

* 2 F_1 double infected females were selected to establish the wcL line.
